# Monthly Summary

**Published:** 1867-05

**Authors:** 


### MONTHLY SUMMARY.

*Blood Corpuscles.*—Different opinions have been expressed by recent microscopical observers upon the constitution of these bodies. Thus Ofsiannikof says that they possess an investing membrane or proper cell-wall, which re-acts differently from the contents. He has observed that crystallization occasionally occurs in the corpuscles while still floating in their own serum, and thinks that many of the changes in form, attributed to the influence of certain reagents, really occur within the corpuscles.

Schultze, however, examining blood which was kept on a peculiar slide at its own temperature, comes to the conclusion that the corpuscles are destitute of investing membrane. Of white corpuscles, he found some smaller than the red ones, some of equal size, and the rest of the typical form. These last exhibited lively and vigorous movements. He belives them too to be destitute of an investing membrane. Their movements cease after two or three hour's exposure to a heat of 100° F. Above this point they soon die, but keep active for twenty hours at 32°.

The red corpuscles exhibit no movement, but when heated to 125° they changed form, becoming indented at the margins, and then detaching rounded fragments, at first with pedicle. At last only minute bodies remained in the blood liquid, the largest being always smaller than unaltered blood-discs and the smallest quite minute granules. He found always small spherical blood-globules, the number of which varied according to the hour of the day, and the significance of which remains undetermined. In violent fevers, the blood-discs always have a tendency to become globular.

---

*Rapidity of Nerve Action.*—Haller attempted, in reading the Æ neid aloud, to count the number of letters he could pronounce in a minute. Finding that he could pronounce 1.500, among which the R, according to his statement, requires ten successive contractions of the stylo-glossus, he affirms that a muscle can contract and relax itself 15000 times in a minute; and as the time of relaxation is as long as that of contraction, each contraction requires about 1-30000 of a minute, or 1-500 of a second. From this Haller concludes that the nervous agent requires the 1-500 of a second to go from the brain to the stylo-glossus muscle. Following out this suggestion, Bois-Reymond has invented an ingenious instrument called a myographion, which registers the contraction of a muscle and the time intervening between that and the primary excitation of the nerve upon which the contraction depends. On applying this apparatus to the sciatic nerve and the gastrocnemius muscle of a frog, it was found that for two inches of nerve, contraction took place in the 1-450 of a second. Hence it is inferred that nerve-force is transmitted at the rate of 75 feet a second.

But does this really measure the rapidity of transmission of nerve-force? May it not as well represent the rate of contraction of muscular fibre, or perhaps a quantity composed of both these factors?

---

*New Surgical Bandage for Fractures.*—The starch apparatus has long been a favorite application to fractures. For starch as a thickening and solidifying material, M. Velpeau has recently suggested soluble glass. It possesses many advantages over anything else yet used for the purpose. It is rigid, affording a much stronger supporter than any of its predecessors, is easily applied, and very neat and clean, it hardens in two or three hours, and can be readily removed by softening with water in which it is soluble at any temperature. It is now quite generally used, both in Europe and this Country. In Baltimore it is extensively made of uniform composition, as one of the ingredients in the manufacture of artificial stone, and every one who desires to use it can easily obtain it at a moderate price, and of any consistence they may desire.

---

*Marriage Between Blood Relations.*—Dr. A. Voisin, Physician to the Bicetre Hospital in Paris, has contributed to the Memoirs of the Society of Authropology a memoir on this interesting subject, in which he takes opposite ground to that usually occupied by medical writers on this question. He made his inquiries in the little French village of Batz, in which the whole populatlon is more or less related by ties of blood. Rejecting all but the intermarriages between persons nearly allied, he publishes the results of his inquiries with the results of 46 marriages, 5 between first, 31 between second, and 10 between fourth cousins. In these he finds none of the unhappy consequences so eloquently described by other writers. There were among the offspring of these marriages no malformations, no mental disorders, no idiocy, cretinism, albinism deafness, dumbness, nor epilepsy, Scrofula existed in but one young girl. Barrenness is almost unknown. Two only, third cousins, were childless, and the other 44 pair had 174 children, an average of about four to each couple.

The children were lively, cheerful, active and more than usually intelligent. The inhabitants of the village are usually long-lived, preserving to the end a good degree of bodily and mental vigour.

The conclusion he arrives at is that where no tendency to hereditary disease or morbid diathesis exists, the marriage of cousins does not deteriorate but rather improves the race.

---

In the Mariana Florida Courier, we find the following account of an apparatus constructed by Dr. T. W. Hentz of that place, for which he deserves great credit.

Mr. Milton Mosely of this county was wounded during the war in his face, carrying away his entire upper lip, and nearly his entire nose/ his palate was cleft its whole length and all the front teeth carried away, making his appearance as unseemly as possible, and interfering with his speech and respiration, to such an extent as to be extremely annoying. Dr. Hentz made for him a palate and teeth, an india rubber nose, supported at its base by wires attached to the plate, and at its upper extremity by a pair of spectacles. Also attached a moustache to the upper lip, thus converting Mr. Mosely into quite a handsome man. His difficulty in speaking is removed, and the appliance, unless by close inspection cannot be distinguished from a natural nose and palate. This ingenious contrivance can be removed and put back at pleasure.

